# American Medical Literature

**Published:** 1854-06

**Authors:** 


### AMERICAN MEDICAL LITERATURE.

THE subject of American medical books was two or three times brought up at the late meeting of the Medical Association in St. Louis. One gentleman offered a resolution condemning the practice of professors recommending as text books their own productions; but the resolution was not adopted. Another resolution was adopted, appointing a committee to inquire into the causes which impede the growth of our medical literature, and report to the Association at its next meeting. The subject is one of much interest. No man actuated by proper feelings can be indifferent to the literary reputation of his country. It cannot be denied that for a long time we were in a state of literary vassalage to England and France altogether unworthy of our national character, and in the estimation of very many we are still far from having attained a position of independence, in this respect, becoming us as a people. No one can have forgotten the taunt of the Editor of the Edinburgh Review, put forth a few years ago—"*Who reads an American book?*" Such a question would hardly be asked by any British reviewer now, but we think the time has co.ne when we should rely more upon ourselves and look less abroad for our professional literature, and should certainly exhibit much less sensitiveness than we have shown to the strictures of foreign critics. We incline to no embargoes; we would impose no restrictions upon the traffic in books, and we thank our brethren of other countries for every valuable emanation from their presses. Knowl edge ought to be free, and we cordially welcome every good book that comes to our shores whatever be its birth-place. But are we just to ourselves in remaining so dependent upon the profession of other countries? Such a state of things must exist in the infancy of a people, but we have passed beyond that stage, and it does seem to us that we have arrived at a period when we ought to declare our literary independence and set up for ourselves.

There is everything in the success of our native medical authors to incite to literary effort. Those books which have been written by American physicians have generally met with great favor.

The writings of Dr. Benjamin Rush had a wide popularity, and were as much read by his countrymen as the productions of any cotemporary European writer. Eberle was as successful as any medical author of his day. Edition after edition of his works was called for during his lifetime, and at least one has been published since his death. The first work published by Chapman, his Therapeutics, attracted much attention and was generally read. Dunglison has been eminently successful as an author, and his colleague, Prof. Meigs, has been hardly less so. Few books have had a more steady sale, or been more universally approved by the profession than the "Dispensatory" of Wood & Bache, and a later production of the former has taken its rank among the standard works on the practice of medicine. Prof. Dickson, of Charleston, has produced works which have given him an enviable reputation, and are to be found widely distributed through the libraries of his brethren. Everybody knows how wide was the circulation of Wistar's Anatomy. Dorsey's Surgery soon made its way into most of the medical libraries in the country, and Gibson's Surgery has passed through numerous editions. The writings of Bartlett have met with universal favor, and have been profitable both to the author and his publishers. Horner, Godman, Warren, the Becks, and Dewees have conferred honor upon their country by their labors, and an ample share of praise has been awarded to the productions of their pens. The great work of Dr. Drake, prepared at the expense of so much time and labor, was hailed by his brethren at home as a production which did honor to American medicine, and has received the highest commendation of European critics. Our colleague and friend, Prof. Miller, has abundant reason to be satisfied with his success as an author, few works on obstetrics having been more applauded than his Human Parturition. The success of another colleague has been great enough to satisfy any ordinary ambition. While Craigie's Pathological Anatomy was nearly twenty years reaching a second edition, and Andral's, we believe, has not yet attained to that stage, a second edition of the Pathological Anatomy of our friend, Prof. Gross, was called for in less than six years after the appearance of the first.

All in the course of these writers has not run smooth; there has been enough of censure to take off the curse resting upon him of whom "all speak well." For example, a very small, and very malignant, and somewhat disappointed critic, has lately spoken of the lamented DRAKE as "a babbling historiographer who had spent most of his life in running about the country, collecting its medical gossipry." But these small, malignant critics, prompted as they are by personal spite, injure none but themselves. The profession turns contemptuously upon them and demands, what have they done for the medical literature of their country? Their voices are very feeble amid the general applause. The past and the future are alike full of encouragement and promise for American medical writers.

As to the objection urged against the recommendation by professors of their own works as text-books, we confess that we do not perceive its force. Nothing seems to us more natural than that the book of the teacher should be the one used as a text-book by his pupils. If his book is uot worth reading, it may be doubted whether his lectures are worth hearing. In Germany, we believe, it is the custom of professors to prepare "hand-books" for their students, and we never heard the practice condemned as evincing a want of modesty in those learned men.

